# Oral Diadochokinesis and Potential Associated Factors in Japanese Older Adult Outpatients

**DOI:** 10.3290/j.ohpd.b5866422

**Published:** 2024-12-02

**Authors:** Tin Zar Tun, Kaung Myat Thwin, Sachiko Takehara, Hiroshi Ogawa

**Affiliations:** a Tin Zar Tun Graduate Student, Division of Preventive Dentistry, Department of Oral Health Science, Faculty of Den-tistry and Graduate School of Medical and Dental Sciences, Niigata University, Niigata, Japan; Universi-ty Lecturer, Department of Paediatric Dentistry, University of Dental Medicine, Yangon, Myanmar. Wro-te the manuscript, data collection, data analysis, and interpretation of results.; b Kaung Myat Thwin Assistant Professor, Division of Preventive Dentistry, Department of Oral Health Science, Faculty of Dentistry and Graduate School of Medical and Dental Sciences, Niigata University, Niigata, Japan. Data collection, supervised data analysis, and interpretation and critically revised manuscript.; c Sachiko Takehara Associate Professor, Division of Preventive Dentistry, Department of Oral Health Science, Faculty of Dentistry and Graduate School of Medical and Dental Sciences, Niigata University, Niigata, Japan. De-signed study, data collection, and interpretation of results, and supervised the manuscript.; d Hiroshi Ogawa Professor and Head, Division of Preventive Dentistry, Department of Oral Health Science, Faculty of Dentistry and Graduate School of Medical and Dental Sciences, Niigata University, Niigata, Japan. De-signed the study, interpretation of results, and supervised the manuscript.

**Keywords:** ageing, diadochokinesis, oral motor function, orofacial neuromuscular complex, oral hypofunction

## Abstract

**Purpose:**

This study investigated oral diadochokinesis (ODK) and its associated factors, including age group differences, among Japanese older adult outpatients.

**Materials and Methods:**

A cross-sectional study was conducted with 127 outpatients (≥65 years) receiving dental check-ups (May 2022–February 2023). Oral function was assessed using ODK (pa/ta/ka/) (KENKOU-KUN®), tongue pressure (TPM-01), masticatory performance (gummy jelly), and swallowing function (RSST). Structured interviews measured social engagement (LSNS-16), depression (GDS-15), and cognitive function (MMSE). Statistical analyses included Chi-square tests, t-tests, and linear regression.

**Results:**

Mean ODK values were 6.2 ± 0.7 (/pa/), 6.1 ± 0.8 (/ta/), and 5.6 ± 0.9 (/ka/). Age-grouped differences were found in tongue pressure and ODK /ta/, /ka/. Unadjusted analysis revealed associations between ODK /pa/ and sex, number of remaining teeth, and social engagement. ODK /ta/ was associated with tongue pressure (B: 0.022, 95%CI: 0.008, 0.036), masticatory performance, and swallowing difficulty. ODK /ta/ and /ka/ showed age and sex association. Adjusted regression analysis showed associations between ODK /pa/ and number of remaining teeth (B: 0.028, 95% CI: 0.004, 0.052), ODK /ta/ and tongue pressure (B: 0.021, 95% CI: 0.007, 0.035), masticatory performance (B: 0.095, 95% CI: 0.018, 0.161), and swallowing difficulty (B: –0.679, 95% CI: –1.192, –0.165).

**Conclusions:**

This study reveals ODK’s multifaceted nature, highlighting its relationships with various oral and psychosocial factors. Associations between ODK (pa and ta) and other oral functions suggest that improving ODK could maintain overall oral health and quality of life in older adults. Incorporating ODK assessments into routine dental check-ups should be further assessed.

The relationship between orofacial function and overall health in older adults has gained increasing attention.^14–16, 23, 25^ In 2014, Japan introduced the concepts of ‘oral frailty’ and ‘oral hypofunction’, emphasising the importance of maintaining oral health to extend healthy life expectancy.^14–15^ Oral functional decline can lead to malnutrition and negatively impact overall health, functional ability and cognition.^15–16, 25,29^ Consequently, maintaining optimal oral health, ensuring a balanced diet, and addressing psychosocial well-being may mitigate the likelihood of frailty.^2,23^

Oral motor function, governed by the orofacial neuromuscular complex,^10^ is crucial for various oral activities. Oral diadochokinesis (ODK) is widely used to assess tongue–lip motor function by measuring the syllable repetition rate (pa, ta, ka).^5^ ODK, through the assessment of repetitive sound production, provides an estimate of neuromotor integration, thereby offering insights into the functionality of the oral neuromotor complex.^8^ It is crucial in evaluating oral hypofunction and potential oral frailty in older adults. ODK impairments can manifest due to a variety of underlying factors, including structural abnormalities, motor dysfunction, sensory deficits, and cognitive impairments.

Identifying older adults with poor oral function is crucial, as it correlates with physical fitness, age, and sex.^31^ Ageing negatively impacts oral functions such as tongue movement, swallowing, and speech, with older adults showing lower ODK performance due to changes in neural control and muscle degeneration.^1^ While the tongue can compensate for missing teeth, age-related declines in labial and lingual muscles reduce masticatory efficiency, affecting daily activities.^11^

Research has consistently shown an association between oral health and social engagement.^34^ However, the relationship between ODK, an essential oral motor function measure, and social disengagement is incompletely understood. Since effective communication depends on proper oral motor function,^4^ ODK declinations could impair speech and social interactions, which leads to social disengagement. This is incorporated by longitudinal studies demonstrating that poor oral health can result in functional limitations and a decline in oral-health-related quality of life, potentially restricting social activities,^22^ and negatively affecting psychological well-being.^19^ While studies have explored ODK in community settings,^11,12, 31^ there are still gaps for further research in the outpatient population.^5^ Outpatients have a higher prevalence of chronic diseases, which might affect ODK performance.^28^ They also benefit from frequent interactions with healthcare professionals, potentially facilitating early detection and intervention for oral health-related issues.

This study focuses on Japanese outpatients, addressing a gap in understanding how ODK relates to other oral functional and psychosocial factors in this population. By targeting outpatients, the research aims to facilitate early interventions to prevent or delay the progression of oral frailty. The study explores how ODK relates to oral function, psychosocial factors, and age-related changes in older Japanese outpatients. We hypothesise that ODK would be associated with other oral functional factors and additional factors such as social engagement, depression, and cognitive function. By improving our knowledge of the oral function of the older adult population, this study potentially leads to more thorough models of healthy ageing that integrate oral health and social factors. Utilising the outcomes of this study in the long-term cohort, regular ODK assessments in outpatient settings could be integrated into routine health check-ups for older adults, promoting a more comprehensive approach to geriatric healthcare.

## MATERIALS AND METHODS

### Study Design and Participants

This cross-sectional study recruited 135 Japanese adults, aged 65 years or older, who visited the Preventive Dental Clinic at University Dental Hospital’s outpatient department for oral health maintenance and examinations from May 2022 to February 2023. Of these older individuals, 127 (94.0%) responded and underwent instrumental and objective assessments. Eight were excluded based on exclusion criteria. Exclusion criteria encompassed incomplete information (4 persons), a history of facial paralysis (1 person), or refusal to participate in either oral function or oral examinations (3 persons). For the age distribution, the participants were divided into two groups: 65–74 years and ≥ 75 years, according to the Joint committee of the Japan Gerontological Society and the Japan Geriatrics Society.^18^ All procedures involving study participants were approved by the Ethics Committee (Reg no. 2021/0172), in compliance with the 1964 Declaration of Helsinki, and each participant provided informed written consent.

### Data Collection

The participant’s age in years (65–74 or 75–90), sex, and education (high school or higher, middle school or lower) were asked. The current study included a questionnaire survey using instrumental tools, oral examination, and oral function assessment (objective measures).

#### Instrumental tools

The study investigated how social engagement, depression, and cognitive function might affect ODK in older Japanese outpatients. An experienced native dentist used interview-style three validated questionnaires, all in Japanese. The Lubben Social Network Scale-6 measured social engagement, with scores below 12 indicating low engagement.^13^ The Geriatric Depression Scale 15 assessed depression risk, with scores of 5 or more suggesting potential depression.^24,26^ The Mini-Mental State Examination evaluated cognitive function, with scores below 24 indicating possible cognitive impairment.^20^

#### Objective assessments

An experienced dentist with a trained assistant dentist conducted all clinical oral examinations and counted the total number of remaining teeth. A trained assistant dentist measured each participant’s height in metres and weight in kilograms and body mass index (BMI) was computed.

Another experienced dentist and a trained assistant dentist assessed ODK using a digital measuring device (KENKOU-KUN®; Takei Scientific Instruments Co., Niigata, Japan).^5^ Participants were instructed to repeat each word (pa/ta/ka) as fast and accurately as they could within 5 s and measured three times. The average value per second was calculated for each syllable.

A tongue pressure measuring device (TPM-01) (JMS, Hiroshima, Japan) was used to measure tongue pressure at the midline of the dorsum of the tongue.^33^

Masticatory performance was evaluated using gummy jelly (UHA Mikakuto Co., Osaka, Japan) and a visual scoring method. Participants were instructed to chew a gummy jelly freely 30 times, and after chewing, to expectorate all the chewed fragments onto a piece of gauze spread over a paper cup. Participants with dentures were instructed to wear them while chewing.^9^ The pieces of gummy jelly were spread out and the investigators subjectively evaluated them by the visual scoring method, which categorises the pieces into ten levels (0–9).

The repetitive saliva-swallowing test^1^ was used to evaluate swallowing function. Participants were instructed to swallow saliva as many times as possible within EC, while the degree of deglutition was determined by the palpation of the larynx.^15,21^ Dysphagia was noted if the participants met the criterion of occurring less than three times per 30 s.^21^

### Statistical Analysis

The Kolmogorov–Smirnov test was used to test for normality. The findings are reported by means and standard deviations, or frequencies and proportions. To assess the relationship between covariate variables and outcomes, the Chi-square test or t-test was used. The correlation coefficients between the variables were calculated to assess the multicollinearity by assessing variance inflation factors (VIF) among the independent variables. The covariables with the dependent variable of ODK were subjected to linear regression analysis. All data were analysed using IBM SPSS Statistics 25 (IBM, New York, USA) with a significance level of less than 5%.

## RESULTS

### Study Participants

Of 127 participants, the mean age is 75.9 ± 6.0 years, and 87 (68.5%) were women. The ODK tests showed mean values of 6.2 ± 0.7 for /pa/, 6.1 ± 0.8 for /ta/, and 5.6 ± 0.9 for /ka/, with the latter falling below the suggested threshold of 6.^5^ Participants had an average of 24.0 ± 4.9 remaining teeth and a masticatory performance of 5.6 ± 0.9. Most of the participants have normal swallowing function (93.7%). While most oral factors were within normal ranges, tongue pressure measurements were lower than expected, with a mean of 28.9 ± 9.3.

### Distribution of Participants According to Age Group

Table 1 stratifies the participants’ characteristics into two age groups: pre-old (65–74 years) and old (≥75 years). Both groups were female predominant. In the 65–74-year age group, all oral factors were within normal limits, except that the mean value for ODK/ka/ was 5.8 ± 0.8, slightly below the normal threshold. Statistical analysis revealed significant differences between age and several oral factors: tongue pressure (p = 0.003), ODK /pa/ (p = 0.001), and /ta/ (p = 0.024).

**Table 1:** Distribution according to age groups

	Total	65–74 years	≥ 75 years	p value
Sociodemographic				
Sex				
Male	40 (31.5)	18 (31.0)	22 (31.9)	0.918
Female	87 (68.5)	40 (69.0)	47 (68.1)	
Education				
high school or higher	70 (55.1)	31 (53.4)	39 (56.5)	0.729
middle school or lower	57 (44.9)	27 (46.6)	30 (43.5)	
Marital status				
married	120 (94.5)	54 (93.1)	66 (95.7)	0.531
unmarried	7 (5.5)	4 (6.9)	3 (4.3)	
BMI	22.0 ± 2.8	21.7 ± 2.4	22.2 ± 3.1	0.314
Instrumental tools				
LSNS				
< 12	48 (37.8)	17 (29.3)	31 (44.9)	0.071
≥ 12	79 (62.2)	41 (70.7)	38 (55.1)	
GDS15				
< 5	96 (75.6)	43 (74.1)	53 (76.8)	0.727
≥ 5	31 (24.4)	15 (25.9)	16 (23.2)	
MMSE (Ref:>24)				
≥ 24	124 (97.6)	56 (96.6)	68 (98.6)	0.460
< 24	3 (2.4)	2 (3.4)	1 (1.4)	
Oral factors				
Number of remaining teeth	24.0 ± 4.9	24.6 ± 4.6	23.5 ± 5.2	0.215
Tongue pressure	28.9 ± 9.3	31.5 ± 9.0	26.7 ± 9.0	0.003
Masticatory performance	5.6 ± 1.7	5.6 ± 1.8	5.6 ± 1.6	0.983
Swallowing function				
≥ 3	119 (93.7)	55 (94.8)	64 (92.8)	0.632
< 3	8 (6.3)	3 (5.2)	5 (7.2)	
ODK/pa/	6.2 ± 0.7	6.3 ± 0.6	6.1 ± 0.7	0.058
ta/	6.1 ± 0.8	6.3 ± 0.7	5.9 ± 0.8	0.001
ka/	5.6 ± 0.9	5.8 ± 0.8	5.4 ± 1.0	0.024
Data are presented as the number of participants (%) or mean ± SD. The significance level is <0.05 using t-test or Chi-square tests. The distribution of variables across the age groups. BMI – body mass index, LSNS – Lubben’s social network scale, GDS – geriatric depression scale, MMSE – mini-mental state examination.

### Association of ODK and Its Potential Associated Factors

Tables 2 and 3 present the results of linear regression analyses for ODK, with multivariable analyses adjusted for sociodemographic characteristics and BMI. The VIF values for all predictors ranged from 1.01 to 1.14, indicating that multicollinearity was not a concern in the model.

**Table 2:** Unadjusted linear regression analysis across ODK

	pa	p value	ta	p value	ka	p value
B (95%CI)	B (95%CI)	B (95%CI)
Sociodemographic
Age (years) (Ref: 65–74)
≥ 75	–0.227 (–0.462, 0.008)	0.058	–0.464 (–0.724, –0.210)	0.001	–0.367 (–0.685, –0.049)	0.024
Sex (ref: Male)
Female	0.277 (0.026, 0.527)	0.031	0.352 (0.067, 0.637)	0.016	0.690 (0.364, 1.016)	<0.001
Education (Ref: high school or higher)
Middle school or lower	0.083 (–0.155, 0.321)	0.489	–0.041 (–0.313, 0.232)	0.768	–0.123 (–0.448, 0.201)	0.453
Marital status (Ref: married)
Unmarried	0.385 (–0.130, 0.900)	0.141	0.382 (–0.208, 0.972)	0.202	0.203 (–0.505, 0.911)	0.571
BMI	–0.026 (–0.068, 0.017)	0.235	–0.040 (–0.088, 0.009)	0.106	–0.039 (–0.097, 0.018)	0.178
Oral factors
Number of remaining teeth	0.026 (0.003, 0.049)	0.030	0.018 (–0.008, 0.044)	0.168	0.011 (–0.02, 0.042)	0.484
Tongue pressure	0.007(–0.006, 0.02)	0.313	0.021 (0.007, 0.035)	0.004	0.008 (–0.009, 0.025)	0.378
Masticatory performance	0.058 (–0.012, 0.127)	0.103	0.090 (0.014, 0.166)	0.021	0.050 (–0.041, 0.141)	0.278
Swallowing difficulties	–0.275 (–0.743, 0.192)	0.246	–0.663 (–1.172, –0.154)	0.011	–0.296 (–0.907, 0.315)	0.340
Instrumental tools
LSNS (Ref: <12)	0.221 (–0.016, 0.459)	0.068	0.056 (–0.212, 0.323)	0.681	0.017 (–0.298, 0.331)	0.917
GDS15 (Ref: <5)	0.190 (–0.076, 0.456)	0.160	0.683 (–0.156, 1.522)	0.504	0.254 (–0.093, 0.601)	0.150
MMSE (Ref: >24)	0.466 (–0.292, 1.225)	0.226	0.015 (–0.440, 0.470)	0.110	0.700 (–0.288, 1.687)	0.163
	pa	p value	ta	p value	ka	p value
Coefficients (B) – unstandardised beta coefficient. Bold represents statistically significant. The significance level is <0.05. BMI – body mass index, LSNS – Lubben’s social network scale, GDS– geriatric depression scale, MMSE– mini-mental state examination. Ref: reference group.

In the unadjusted linear regression analysis, we observed some significant associations. The number of remaining teeth shows a positive linear association with ODK /pa/ (B: 0.027, 95% CI: 0.003, 0.051). Tongue pressure was positively associated with ODK /ta/ (B: 0.022, 95% CI: 0.008, 0.036). Sex was significantly associated with all ODK measures. Age demonstrated a negative association with ODK /ta/ (B: –0.464, 95% CI: –0.724, –0.210) and /ka/ (B: –0.367, 95% CI: –0.685, –0.049). ODK /pa/ was associated with LSNS (B: 0.301, 95% CI: 0.063, 0.540), while ODK /ta/ showed an association with MMSE (B: 0.892, 95% CI: 0.014, 1.770). Notably, ODK /ka/ exhibited no significant relationships with variables other than age and sex.

We conducted an adjusted linear regression analysis to examine the factors associated with ODK. We adjusted sociodemographic characteristics: age and sex, education, and BMI, and the analysis was performed in steps, with each step adding a new independent predictor to the adjusted model. The findings from the adjusted linear regression analysis corroborated and refined associations. The number of remaining teeth maintained a significant positive association with ODK /pa/ (B: 0.028, 95% CI: 0.004, 0.052). Likewise, tongue pressure demonstrated a positive relationship with ODK /ta/ (B: 0.021, 95% CI: 0.007, 0.035). Furthermore, we found a positive linear association between ODK /ta/ and masticatory performance (B: 0.095, 95% CI: 0.018, 0.161), as well as a negative association with swallowing difficulty (B: –0.679, 95% CI: –1.192, –0.165).

## DISCUSSION

The current study focused on outpatients visiting a dental hospital for regular maintenance check-ups since they are easy to access for follow-up examinations, and outpatient assessments could facilitate the implementation of early interventions to prevent or delay the progression of oral frailty. To the best of our knowledge, this study represents the first endeavour to evaluate ODK in outpatients undergoing routine dental examinations at hospital-based preventive dental clinics. Furthermore, it is distinctive in assessing ODK’s association with oral, social, and cognitive factors. Our findings reveal statistically significant correlations between specific ODK measures (/pa/ and /ta/) and select oral and psychosocial variables. Notably, we observed no significant associations between the ODK measure /ka/ and the oral and psychosocial parameters examined in this investigation.

Regarding orofacial functional factors, we found that the number of remaining teeth, tongue pressure, and masticatory performance influenced ODK performance for /pa/ and /ta/ syllables. Our regression model indicated a significant association between ODK /pa/ and the number of remaining teeth. Additionally, ODK /pa/ was associated with sex, and social engagement, corroborating previous findings on the relationship between ODK and social interaction.^19^ Communication disorders in older adults, such as voice and hearing loss, are linked to disengagement from social networks, reduced social support, and decreased social participation.^19^ Poor lip and tongue movement may adversely affect speech and mastication, potentially leading to stress-induced weakness and social withdrawal, particularly in individuals over 70 years of age.^14^ This finding implies with the previous research report that poor oral health can lead to reduced social interactions due to issues like difficulty speaking, eating in public, or low self-esteem related to oral health since ODK provides insights into the speed and accuracy of oral movements crucial for speech and swallowing.^27,22^ The bidirectional association between oral health and social isolation has also been reported.^34^

Our analysis indicated that ODK /ta/ performance was influenced by age, sex, tongue pressure, and cognitive status. The regression model9 highlighted a robust association between ODK /ta/ and tongue pressure, consistent with earlier research.^24^ The articulation of the /ta/ sound involves brief contact between the tongue tip and the anterior palate, requiring upward tongue movement.^3^ This motion closely resembles the mechanism of tongue pressure generation, which involves lingual elevation and mouth floor raising through hyoid bone elevation, driven by suprahyoid muscle activation.^7^ Additionally, the observed connection between ODK performance and cognitive function aligns with previous studies suggesting that mild cognitive impairment may affect ODK.^12^

Regarding ODK /ka/, our findings revealed correlations with age and sex, but not with other examined factors. This partially corroborates previous research indicating that ODK /ka/ tends to exhibit a more pronounced age-related decline.^6^ While some studies have associated ODK /ka/ with swallowing difficulties,^17^ it is important to note that dysphagia arises from various factors, including reduced lingual strength and mobility.^3^ Interestingly, our study did not demonstrate an association between ODK /ka/ and swallowing disorders. This discrepancy may be attributed to differences in study populations; unlike previous research focusing on community-dwelling individuals with dysphagia, our study participants appeared to have normal swallowing function, potentially explaining the lack of association observed with ODK /ka/.

The results revealed age group differences in orofacial muscle functions, particularly in ODK performance for /ta/ and /ka/ syllables, as well as in tongue pressure. The mean values for ODK /pa/ and /ta/ exceeded the established normal thresholds, while the mean for /ka/ was marginally below this threshold. Notably, all ODK values in our study were superior to those reported in previous studies focusing on outpatient populations.^5^ The observed age group differences in ODK performance among older adults corroborate existing literature.^31^ This phenomenon may be attributed to alterations in neural control mechanisms and biomechanical changes, including the degeneration of muscle tissue and connective structures.^1^ These findings align with research indicating that muscular degeneration initiates around age 25 and progresses over the lifespan^32^ and suggest that age may play a role in certain oral function declinations, particularly those related to tongue movement and pressure. Additionally, there is evidence that proper evaluation of oral function and proper oral function exercises are associated with physical fitness among older adults.^23^ Thus, timely evaluations and appropriate exercises during dental check-ups could contribute to healthier living and an enhanced quality of life for older adults.^23^

This study provides important insights into the oral function of older adults by recruiting participants from outpatient settings and strengthening by evaluating objective assessments of oral function. The observed declinations in ODK performance highlight a crucial area for dental practitioners’ attention. Future research should evaluate discrepancies among ODK components (pa/ta/ka) and develop tailored therapeutic approaches. The outcomes of this study could potentially lead to the integration of regular ODK assessments in outpatient settings as part of routine health check-ups for older adults, promoting a more comprehensive approach to geriatric healthcare.

This research investigates the distinct relationships between ODK components and social engagement in older adults, aiming to provide new insights into oral function and social engagement in ageing populations. These findings could contribute to more comprehensive models of healthy ageing integrating oral health and social factors. Our findings highlight the potential of using ODK as a diagnostic tool in preventive dentistry. Although there are some limitations, such as a small sample size, single-site recruitment, and a cross-sectional study design, the results have implications for enhancing oral health assessments and interventions in geriatric dentistry. The high response rate (94.1%) and transparently reported sampling method partially mitigate sampling bias, though generalizability may be limited.

However, the results may not be fully generalisable because the study was conducted at a single site with convenience sampling, and it did not consider factors like dental caries and periodontal status, which could affect the accuracy of the findings. These might result in under- or overestimation of certain relationships. A large scale that involves multiple sites, with a longitudinal study design would likely yield a more comprehensive, generalisable, and nuanced understanding of ODK and its associations with oral health factors in older adults.^30^ Future longitudinal studies could help establish causal relationships between ODK measures and oral health outcomes, track oral frailty progression, evaluate early intervention effectiveness, and reveal stronger associations over time. This research could advance the field, leading to improved care approaches and a deeper understanding of oral health in older adults.

In conclusion, our results demonstrate the impact of ageing on orofacial muscle function, with higher ODK values observed in our study population suggesting potential distinctions between individuals seeking preventive care and those in other clinical settings. This study emphasises the complexity of oro-motor function in older adults and the need for more comprehensive, longitudinal research to fully understand ODK’s role in geriatric oral health assessment and intervention.

Special thanks are extended to Dr Noburo Kaneko, Dr Kumiko Minagawa, Dr Masateru Watanabe, and all the colleagues and staff from the Division of Preventive Dentistry. The authors would like to thank all our participants who took time and great effort to participate.

### Funding

This study was partially supported by the Futoku Foundation and by the JSPS KAKENHI Grant-in-Aid for Scientific Research (C), JP19K10439. The funder has no role in the study design, data collection, analysis, or interpretation of data or in writing the manuscript.

### Ethical considerations

All procedures involving study participants were approved by the Niigata University Ethics Committee in compliance with the 1964 Declaration of Helsinki (2021/11/10) (Reg no. 2021/0172), and each participant provided informed written consent.

### Participant consent statement

All the participants provided informed written consent for all the steps and procedures that the research will perform. They have the right to withdraw at any point in the procedure.

### Data availability

Because of ethical considerations, we could not provide the discrete data. However, the data are available upon request to the corresponding author.

## REFERENCES

**Table 3:** Adjusted linear regression analysis of ODK of the participants

Variables	pa	p value	ta	p value	ka	p value
B (95%CI)	B (95%CI)	B (95%CI)
Oral factors						
Number of remaining teeth	0.028 (0.004, 0.052)	0.020	0.020 (–0.006, 0.047)	0.129	0.013(–0.018, 0.045)	0.394
Tongue pressure	0.009(–0.005, 0.022)	0.199	0.021 (0.007, 0.035)	0.004	0.009 (–0.008, 0.026)	0.294
Masticatory performance	0.061 (–0.010, 0.131)	0.093	0.095 (0.018, 0.161)	0.016	0.054 (–0.038, 0.146)	0.247
Swallowing difficulties (Ref: >2)	–0.293 (–0.770, 0.184)	0.226	–0.679 (–1.192, –0.165)	0.010	–0.315 (–0.934, 0.304)	0.316
Instrumental tools						
LSNS (Ref: <12)	0.227 (–0.017, 0.472)	0.068	0.037(–0.236, 0.309)	0.790	0.009(–0.312, 0.330)	0.957
GDS15 (Ref: <5)	0.180(–0.092, 0.452)	0.193	0.080 (–0.221, 0.381)	0.599	0.238(–0.115, 0.590)	0.185
MMSE (Ref: >24)	0.540(–0.230, 1.309)	0.168	0.756 (–0.087, 1.598)	0.078	0.781(–0.215, 1.777)	0.123
Adjusted for age, sex, education, BMI and respective independent variables using adjusted linear regression analysis. Coefficients (B) resulting from the regression analysis are expressed as the difference in tongue–lip motor function due to a change of one unit in each independent variable. Coefficients for categorical variables are compared to the reference category indicated by B equal to 0. Ref means reference group. The significance level is <0.05, marked in bold. LSNS – Lubber’s Social network scale, GDS – geriatric depression scale, MMSE – mini-mental state examination.
